# Enhanced Soft 3D Reconstruction Method with an Iterative Matching Cost Update Using Object Surface Consensus

**DOI:** 10.3390/s21196680

**Published:** 2021-10-08

**Authors:** Min-Jae Lee, Gi-Mun Um, Joungil Yun, Won-Sik Cheong, Soon-Yong Park

**Affiliations:** 1 Graduate School of Electronics and Electrical Engineering, Kyungpook National University, Daegu 41566, Korea; suddenly90@naver.com; 2 Digital Broadcasting Research Division, Electronics and Telecommunications Research Institute, Daejeon 34129, Korea; gmum@etri.re.kr (G.-M.U.); sigipus@etri.re.kr (J.Y.); wscheong@etri.re.kr (W.-S.C.)

**Keywords:** stereo vision, multi-view stereo matching, iterative, refinement, view synthesis

## Abstract

In this paper, we propose a multi-view stereo matching method, EnSoft3D (Enhanced Soft 3D Reconstruction) to obtain dense and high-quality depth images. Multi-view stereo is one of the high-interest research areas and has wide applications. Motivated by the Soft3D reconstruction method, we introduce a new multi-view stereo matching scheme. The original Soft3D method is introduced for novel view synthesis, while occlusion-aware depth is also reconstructed by integrating the matching costs of the Plane Sweep Stereo (PSS) and soft visibility volumes. However, the Soft3D method has an inherent limitation because the erroneous PSS matching costs are not updated. To overcome this limitation, the proposed scheme introduces an update process of the PSS matching costs. From the object surface consensus volume, an inverse consensus kernel is derived, and the PSS matching costs are iteratively updated using the kernel. The proposed EnSoft3D method reconstructs a highly accurate 3D depth image because both the multi-view matching cost and soft visibility are updated simultaneously. The performance of the proposed method is evaluated by using structured and unstructured benchmark datasets. Disparity error is measured to verify 3D reconstruction accuracy, and both PSNR and SSIM are measured to verify the simultaneous enhancement of view synthesis.

## 1. Introduction

The 3D depth data are widely used in many applications such as object detection, autonomous driving, 3D SLAM, and light-field, etc. The 3D depth of real objects can be obtained by several methods, such as the structured-light, stereo vision, ToF, and LiDAR methods, etc. Among these, stereo vision is one of the most common techniques that can acquire high-resolution 3D depth maps [1,2,3]. Multi-view stereo vision has the advantage of obtaining accurate and dense depth (disparity) information using many cameras from different viewpoints. However, it is still difficult to obtain perfect 3D depth maps, even when using multiple cameras, due to many inherent stereo vision problems such as occlusion, light reflection, and textureless objects. 

Multi-view stereo vision can be used also for novel view synthesis. In a view synthesis technique, a new image is generated from a novel view using the depth and color of neighboring views (in the geometry-based view synthesis). Therefore, the depth accuracy is very important in view synthesis because the pixel color in the new image is determined by the depth of the neighbor views. Penner et al. [4] propose a view synthetics method that is based on the occlusion-aware depth obtained by a soft 3D reconstruction method (Soft3D). In [4], the authors introduce a volumetric space called soft visibility as the probability measure of the voxel visibility from the views of all cameras. The soft visibility is mainly used to iteratively update the inverse depth (disparity) of occlusion areas. A summary of the Soft3D method is as follows. When provided with the multi-view images and camera external poses, the pairwise matching costs between a reference view and its neighbor views are computed using the Plane Sweep Stereo (PSS) algorithm [5,6]. Next, the PSS matching costs are integrated to generate the disparity map of the reference view. After the disparity maps of all the reference views are generated, they are used to compute the soft visibility of all views. The soft visibility and the PSS matching costs are then used to generate new disparity maps. With this closed-loop scheme, the soft visibility is updated iteratively.

However, the Soft3D method has an inherent problem because only the soft visibility is updated. The final disparity map of a reference view is determined by the weighted averaging of the soft visibility and the PSS matching costs. Because the original PSS matching costs contain incorrect values due to occlusion, color noise, a textureless surface, speckle reflex, etc., they continuously affect the disparity map generation during the refinement process. One method to solve this problem is to use accurate-guided depth data which can be obtained from an additional depth sensing device, such as LiDAR sensors [7]. This method refines the incorrect matching costs using sparse-guided depth data. However, this method requires additional processes such as the calibration between the camera and external device.

In this paper, we propose a new multi-view, stereo matching pipeline called EnSoft3D. The goal of the proposed method is to obtain high-quality depth maps by improving the accuracy of PSS matching costs. Our main contributions are summarized as follows:

(1) A new cost update process is introduced in the pipeline of EnSoft3D to simultaneously refine the PSS matching costs and soft visibility of multiple views.
(2) The PSS matching cost is refined using the object surface consensus. The object surface is determined in sub-pixel accuracy from the surface consensus volumes.
(3) An inverse Gaussian kernel is derived from the object surface. Then, the kernel is used to minimize the PSS matching costs around the surface.

After several update iterations, the PSS costs of both the surface and occlusion pixels are simultaneously improved. The final disparity maps are generated using the updated PSS cost and soft visibility.

**Figure 1:**
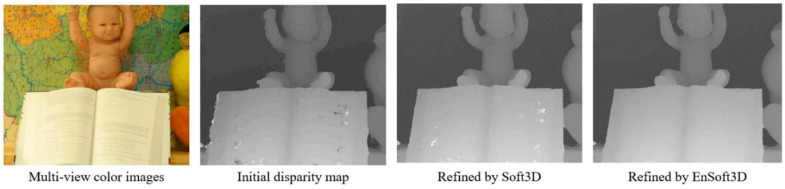
Disparity results using structured multi-view images from *Middlebury 2006* (*Baby2*). Unlike Soft3D, which calculates only visible probability using initial matching costs, EnSoft3D updates the matching costs using object surface probability. It is possible to obtain an accurate disparity map because both the matching cost and the visible probability are refined simultaneously.

An example in Figure 1 shows that the proposed method generates a more accurate depth compared with the result of Soft3D. The qualitative and quantitative evaluation of the proposed method is completed using multi-view stereo datasets with a small camera baseline (*Middlebury 2003, 2006* [8,9], *ETH3D low resolution* [10], *Fountain-P11* [11]). Another evaluation is completed using the results of the view synthesis. By the qualitative evaluation of the new synthesized images, we show the improved performance of our EnSoft3D method. Evaluation is completed with the measurement of the peak signal-to-noise ratio (PSNR) [12] and the structural similarity index map (SSIM) [13] using the *Middlebury* dataset.

The content of our paper is as follows: Section 2 shows the previous studies on the stereo matching technique including the follow-on works of Soft3D; Section 3 describes the base algorithms of the proposed method, PSS and Soft3D; Section 4 describes the details of the proposed method; and Section 5 shows the qualitative and quantitative evaluation of the 3D reconstruction and view synthesis.

## 2. Previous Works

In multi-view stereo vision, the refinement of either the disparity map or matching cost volume is frequently performed for dense and accurate 3D reconstruction. For the disparity map refinement, filtering techniques are commonly applied using a guided filter [14] and weighted median filter [15,16]. However, 2D image filtering techniques have limitations in disparity improvement. In order to refine the disparity map, such filters should be applied to a 3D matching cost volume where an accurate disparity is selected from the refined cost volume.

Hosni et al. [17] propose a cost volume refinement method using the guided filter technique. This method applies a guide filter to each disparity plane of the stereo matching cost volume. The disparity noise is removed and the object edge in the cost volume is preserved by the guided filter. Therefore, this method generates edge-preserved disparity maps. This method is simple and very efficient; however, it is subject to incorrect refinement error due to the color similarity of the guiding image. 

Another cost volume refinement method is Guided Stereo Matching proposed by Poggi et al. [7]. For accurate disparity map generation, this method uses additional 3D LiDAR sensor data. It generates a Gaussian kernel based on the 3D depth from the LiDAR sensor. Then, the cost value is updated using the Gaussian kernel. Because of the accuracy of the LiDAR sensor, it is possible to obtain low-noise disparity maps. However, an additional depth sensor is necessary and the measurement error of the sensor can be included in the disparity map.

Several problems with multi-view stereo vision are solved using deep learning methods. At the early stage of deep learning research, it was only used in some cases, such as for cost computation using deep features and disparity map refinement [18,19,20]. Recently, various deep learning-based methods were introduced for multi-view stereo vision. For example, DeepC-MVS [21] uses a deep network for confidence prediction and DeepPCF-MVS [22] uses a segmentation network for multi-view stereo. In addition, several end-to-end methods are also proposed, such as MVDepthNet [23], DeepMVS [24], and DPSNet [25]. Due to such deep learning investigations, the performance of multi-view stereo matching has greatly increased. However, it is still difficult to obtain a consistent performance in various environments due to the limited training dataset.

Multi-view stereo vision is also used for view synthesis [26]. In the geometry-based view synthesis, the depth acquisition of both occluded and non-occluded areas is very important to synthesize new images in novel viewpoints. The Soft3D method was originally introduced for novel view synthesis [4]. Soft3D introduced a view synthesis pipeline to synthesize accurate images, even in the occlusion areas, by a visibility refinement process. The original work was cited in many follow-on view synthesis investigations [27,28,29]. However, few investigations were introduced to iteratively update the PSS matching costs for the purpose of obtaining accurate 3D depth reconstruction. In addition, due to the recent research trend of using deep learning network for view synthesis, most follow-on investigations employed deep learning networks to refine the volume of PSS matching cost or multiplane images (MPI). Zhou et al. [27] introduce MPI which represents an approximate light field as a stack of semi-transparent, colored depth layers. They use a deep learning network to generate color and alpha images of MPI from the PSS volume of a stereo pair. This approach is similar to Soft3D in that the PSS volume is used as the input of the deep network to decide the weights of blending the background color with the PSS matching features for MPI generation. Flynn et al. [28] introduce a gradient descent deep learning network to iteratively enhance the color and alpha of MPI. The iterative enhancement scheme of the network is similar to the refinement of the soft visibility of Soft3D. Srinivasan et al. [29] introduce a view extrapolation method using the MPI training network. This network employs a 3D convolutional network to generate and train occlusion-free MPI from PSS volumes as network inputs. The generation of occlusion-free MPI is similar to the soft visibility refinement of the Soft3D method.

**Figure 2:**
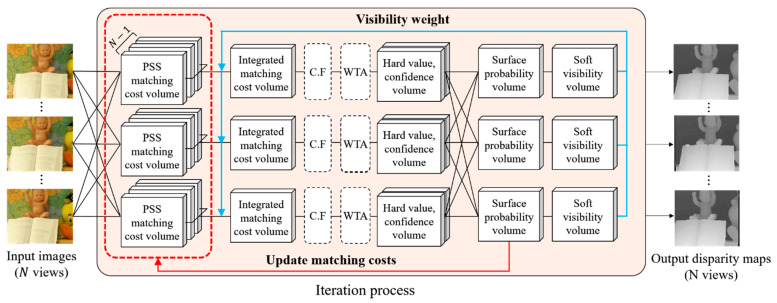
The proposed EnSoft3D multi-view stereo matching scheme. In each reference view, the PSS matching costs are computed using neighbor views and then they are integrated to the reference view. Then, a disparity map is generated from the integrated matching cost volume using cost volume filtering (CF) and Winner-Take-All (WTA). Disparity maps of all views are used to compute the object surface probability and the soft visibility in each view. The PSS matching cost is updated using the consensus volumes and re-integrated with the soft visibility. This closed-loop process is repeated several times to obtain final disparity maps of all views.

In terms of 3D reconstruction, the Soft3D method has an inherent problem in that it does not refine the PSS matching cost volumes. Because the initial PSS matching cost may contain erroneous values, updating only the soft visibility could have inherent limitations for obtaining more accurate depth and view synthesis results. In contrast, we propose a new multi-view stereo matching scheme to simultaneously update both visibility and PSS matching costs. Using the proposed scheme, the matching costs of difficult image areas, such as a textureless surface, color noises, and occluded areas, can be refined better than Soft3D. By improving the 3D reconstruction performance using the proposed method, we can also improve the novel view synthesis performance. The proposed scheme is shown in Figure 2.

## 3. Framework of Soft 3D Reconstruction

The proposed method is developed based on the Soft3D method which was originally introduced for view synthesis. In this section, we briefly describe the 3D reconstruction framework of the Soft3D method. The 3D reconstruction framework consists of two steps, initial disparity map generation and disparity refinement. The initial disparity map is generated using the PSS algorithm. The PSS method is a volumetric, cost-matching, computation method based on projection and back-projection between multi-view images. The PSS algorithm needs a significant amount of computation; however, it has an advantage for estimating an initially accurate disparity map. The second step is disparity refinement. This step computes and refines the soft visibility iteratively. The refined soft visibility is used for obtaining accurate, occlusion-aware disparity maps.

In Soft3D and EnSoft3D, all processes are performed in the disparity space (inverse depth). Therefore, we explain the detailed algorithms in the disparity space. The intrinsic and extrinsic parameters of multi-view cameras are supposed to be calibrated.

### 3.1. Initial Disparity Map Generation

The PSS algorithm computes the matching cost volumes of a reference view using all of the neighbor views. A matching cost volume consists of multiple inverse depth planes which have the same resolution as input image. The number of the inverse depth planes (disparity ranges) is usually defined as the depth range of a reconstruction scene. In addition, the interval between each inverse depth plane is determined by a scale factor s. The relationship between the inverse depth (disparity) d and real depth Z in 3D space is calculated by (1):

(1) $$s=\frac{D\;\cdot \;dist_{min}}{f}\;,\;\;\;Z=\frac{f\;\cdot s}{d}\;,$$

where D is the number of inverse depth planes to be sampled (max disparity), $dist_{min}$ is the minimum depth, and f is the focal length. 

The reference view has N−1 PSS matching cost volumes, which N is the number of input views. Each PSS matching cost volume $V_{r,i}$ contains the matching cost between the reference view r and i-th neighbor view (i∈N−1). As for the cost computation, we use the Sum of Absolute Difference (SAD) [30] and the Census Transform (CT) [31], whose cost is computed as:

(2) $$SAD\left(I_{r}\left(x,y\right),I_{i}\left(x_{i},y_{i}\right)\right)=\frac{\sum_{w}\left|\left(I_{r}\left(x^{w},y^{w}\right)-I_{i}\left(x_{i}^{w},y_{i}^{w}\right)\right)\right|}{n_{w}}\;,$$

(3) $$CT\left(I_{r}\left(x,y\right),I_{i}\left(x_{i},y_{i}\right)\right)=\tau_{adg}\cdot \sum_{w}1(1(I_{r}\left(x^{w},y^{w}\right)<I_{r}\left(x,y\right))\neq 1(I_{i}\left(x_{i}^{w},y_{i}^{w}\right)<I_{i}\left(x_{i},y_{i}\right))),$$

(4) $$V_{r,i}\left(x,y,d\right)=\alpha \cdot SAD\left(I_{r}\left(x,y\right),I_{i}\left(x_{i},y_{i}\right)\right)+\left(1-\alpha \right)\cdot CT\left(I_{r}\left(x,y\right),I_{i}\left(x_{i},y_{i}\right)\right)\;,$$

where $\left(x_{i},y_{i}\right)$ is the pixel coordinate of i-th neighbor view, whose pixel is computed by the perspective projection geometry based on the camera pose; the voxel $V_{r,i}\left(x,y,d\right)$ contains a cost value of between (x,y) and $\left(x_{i},y_{i}\right)$ pixels; and $n_{w}$ is the number of pixels in the pixel window w. SAD computes the color difference between the reference and neighbor views. A matching function 1(condition) in CT is the characteristic function, where its output is 1 if the condition is satisfied, and is otherwise 0. In (3), CT compares the color consistency between the reference and a neighbor view using the hamming distance. The scale of hamming distance is relatively smaller than that of SAD; therefore, we adjust cost importance using $\tau_{adg}$. Two cost computations are completed in a matching window (w). Then, the two costs are integrated by using a user parameter α. In normal experiments, the user parameters are set as follows; α = 0.3, and $\tau_{adj}$ = 5, and *w* is between 3 × 3 and 7 × 7 depending on the input image resolution.

When all PSS matching cost volumes of the reference view are computed, they are all integrated into a new cost volume by simple averaging, which is defined as the initial cost volume of the reference view, as in (5). Then, to remove noisy cost values, an edge-preserving filter ($W_{guide}$, guided filter) is applied to each inverse depth plane in the initial cost volume $V_{r}$, as in (6). Finally, the initial disparity map $D_{r}$ is generated using the Winner-Take-All (WTA, (7)) method:

(5) $$V_{r}^{raw}\left(x,y,d\right)=\frac{\sum_{i\in N-1}V_{r,i}\left(x,y,d\right)}{N-1}\;,$$

(6) $$V_{r}\left(x,y,d\right)=\sum_{\left(\hat{x},\hat{y}\right)\in W_{\mathrm{guide}}}W_{guide}\left(\hat{x},\hat{y}\right)V_{r}^{raw}\left(\hat{x},\hat{y},d\right)\;,$$

(7) $$D_{r}\left(x,y\right)=\underset{d\in D}{argmin}\left(V_{r}\left(x,y,d\right)\right)\;.$$

**Figure 3:**
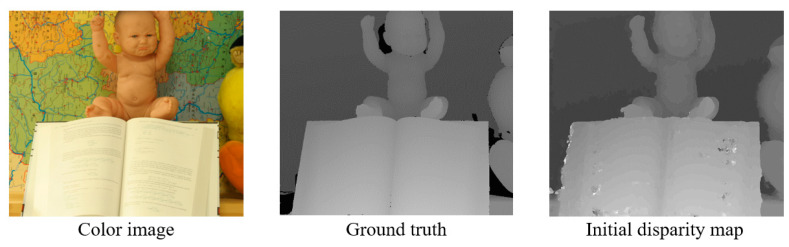
An example of initial disparity map generated by the PSS algorithm (*Middlebury 2006: Baby2*). There are erroneous pixels in the occlusion and textureless areas.

Figure 3 shows an example of the initial disparity map generated by the PSS algorithm. The initial disparity map has erroneous pixels in the occlusion and textureless areas. The Soft3D method refines the disparity in the occlusion area by employing a soft visibility.

### 3.2. Refinement of Disparity Cost Volume

The Soft3D method proposed in [4] computes the soft visibility of all views to refine the disparity of the occlusion area. In Soft3D, ‘Soft’ means that the visibility of a 3D voxel is softly represented by the probability values. Thus, the soft visibility is the visible probability of a voxel in the multi-view images. The soft visibility is computed as follows. When there is an initial disparity map of the reference view r, the vote value ($VoteVal_{r})$ and vote confidence ($VoteConf_{r}$) volumes are generated using (8) and (9). The vote value represents the hard object surface volume and the vote confidence represents the hard visibility volume. Here, ‘hard’ means that those volumes consist of binary values. Therefore, voxels which are supposed to be on the object surface in $VoteVal_{r}$ are set to 1 and the others are set to 0. For the $VoteConf_{r}$ volume, all visible voxels in front of the object are set to 1 and those behind the object are set to 0:

(8) $$VoteVal_{r}\left(x,y,d\right)=\{\begin{array}{cc} 1 & \;d=D_{r}\left(x,y\right)\\ 0 & \;otherwise \end{array}$$

(9) $$VoteConf_{r}\left(x,y,d\right)=\{\begin{array}{cc} 1 & d\geq D_{r}\left(x,y\right)\\ 0 & otherwise \end{array}\;.$$

Once the $VoteVal_{r}$ and $VoteConf_{r}$ volumes of all the views are computed, they are used to compute the consensus volume ($Consensus_{r}$) of each reference view as follows:

(10) $$Consensus_{r}\left(x,y,d\right)=\frac{\sum_{i\in N}VoteVal_{i}\left(x_{i}^{\prime },y_{i}^{\prime },d_{i}^{\prime }\right)}{\sum_{i\in N}VoteConf_{i}\left(x_{i}^{\prime },y_{i}^{\prime },d_{i}^{\prime }\right)}\;.$$

where ($x_{i}^{\prime },y_{i}^{\prime },d_{i}^{\prime }$) are the voxel coordinates, which are the projection of a voxel (x,y,d) of the reference view to the i-th neighbor view. The consensus value ranges from 0 to 1, which can be considered as the surface probability. If the value is close to 1, it means that the voxel has a high probability of belonging to a real object surface. After $Consensus_{r}\left(x,y,d\right)$ is computed, the guide filter is applied to refine the probability using multi-view images. Finally, as in (11), the soft visibility volume $SoftVis_{r}\left(x,y,d\right)$ of the reference view is computed using the cumulative summation of consensus in each pixel ray:

(11) $$SoftVis_{r}\left(x,y,d\right)=max(0,1-\sum_{\hat{d}\in D,\hat{d}>d}Consensus_{r}\left(x,y,\hat{d}\right)).$$

$SoftVis_{r}\left(x,y,d\right)$ represents the visible probability of a voxel in the reference view. Therefore, in an ideal case, voxels in front of the object surface have a value of 1, and the other voxels behind the surface have a value of 0.

The voxels in the occlusion area are not visible, thus their PSS matching costs are most likely erroneous. However, in the PSS algorithm, these erroneous matching costs are integrated with the other costs calculated from the other views. This is one of the reasons that the incorrect disparity is computed in the occlusion areas. To reduce the effect of the erroneous costs of invisible voxels, the Soft3D algorithm uses the soft visibility in the weighted averaging the PSS matching costs to obtain the refined cost volume $V_{r}^{\prime }\left(x,y,d\right)$ as shown in (12):

(12) $$V_{r}^{\prime }\left(x,y,d\right)=\frac{\sum_{i\in N-1}V_{r,i}\left(x,y,d\right)SoftVis_{i}\left(x_{i}^{\prime },y_{i}^{\prime },d_{i}^{\prime }\right)}{\sum_{i\in N-1}SoftVis_{i}\left(x_{i}^{\prime },y_{i}^{\prime },d_{i}^{\prime }\right)}\;.$$

## 4. Enhancement of Soft 3D Reconstruction

In this section, we describe the details of the EnSoft3D method in order to obtain an improved 3D reconstruction performance. Soft visibility ($SoftVis_{i}$) is a key property to decide the integration weight of the PSS matching cost volumes ($V_{r,i}$), between the reference (*r*) and a neighbor view (i), as shown in (12). The Soft3D method iteratively updates the disparity maps using only the soft visibility. However, to enhance the accuracy of the disparity maps, the erroneous PSS matching costs must be improved because $V_{r}\left(x,y,d\right)$ is the combination of the soft visibility and the PSS costs. This is our main motivation for introducing the PSS matching cost update process. 

The proposed update process consists of two steps. The first step is the object surface decision which decides the object surface in each pixel’s ray in the disparity space. The second step is the matching cost update using the decided object surface. By repeating these two steps, the matching costs of the surface voxels become reduced compared to the other voxels.

### 4.1. Object Surface Decision

Multi-view stereo is a very time-consuming and difficult problem because all the matching costs of multiple stereo images must be optimized simultaneously. If there is an external depth sensor which can guide the disparity of some reference views or pixels, matching cost refinement can be completed with the guiding views, as described in Section 1. However, using an external device requires additional processes (coordinate calibration, data conversion), and some device measurement errors can affect the result.

**Figure 4:**
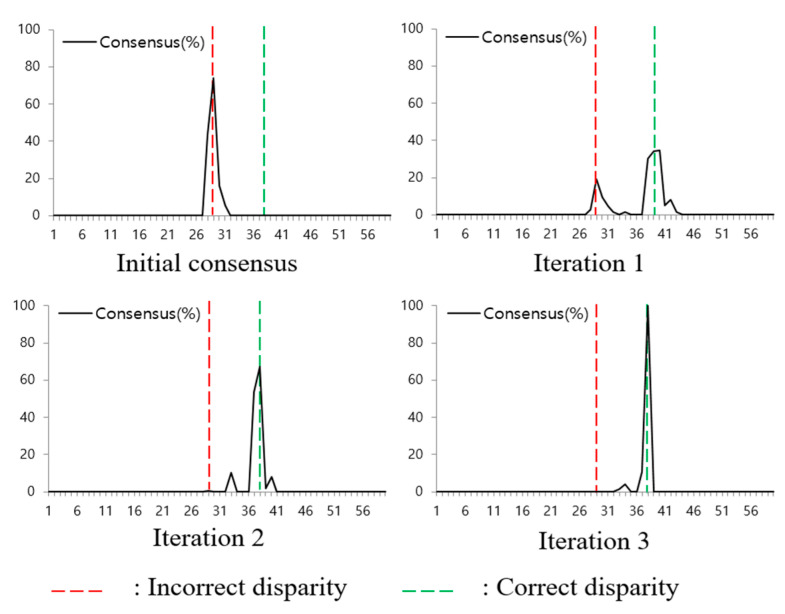
Transition of the depth consensus in consecutive iterations. Red-dotted line is incorrect disparity and green-dotted line is correct disparity.

The Soft3D method uses consensus volumes to compute the soft visibility as shown in (11). A consensus volume is computed in the disparity space, and each voxel value represents its likelihood of belonging to the object surface. Therefore, the consensus is iteratively improved and it has a high value near the object surface. Figure 4 shows an example of the consensus update in an occlusion pixel. In the beginning, the peak consensus is at an incorrect disparity position. However, after several iterations, it is refined and the peak point moves to the correct disparity value. From this consensus update process, we find that the surface consensus can be a reliable property to update the PSS cost volumes.

**Figure 5:**
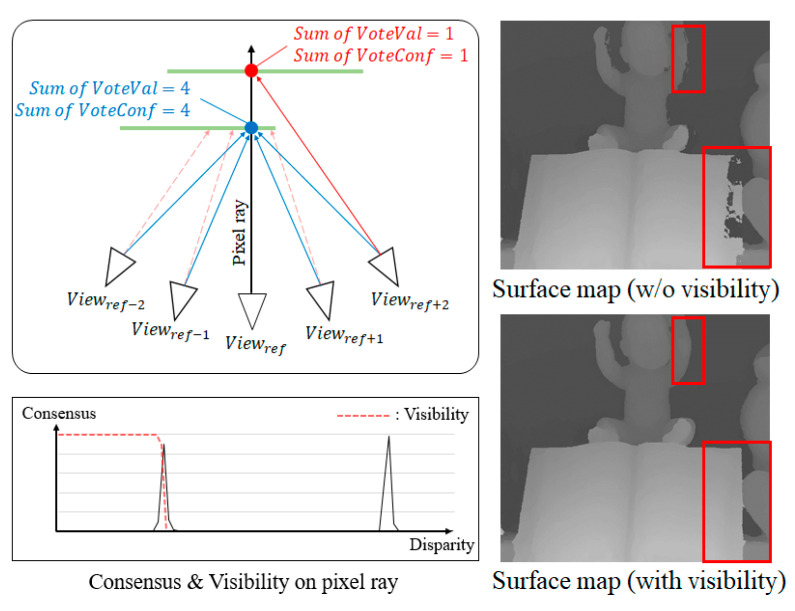
Multiple consensuses in occlusion areas (red box). The matching cost of the occluded areas must be updated by only the visible surface. For this reason, the proposed method decides the object surface by using the consensus with the visibility mask.

However, the object surface is sometimes occluded by another surface as shown in Figure 5 (left). In Figure 5, the blue-colored point is visible from the reference view but the red-colored point is not. On the other hand, both points can be visible from another view as shown in the figure. Even though the red-colored point is not visible from the reference, the surface consensus can also be high at this point because the other neighbor views can see the surface point.

When the surface consensus of the reference view is used to update the PSS matching cost, only visible surfaces should be used. To prevent the consensus of an occluded surface from updating the PSS matching cost, we multiply a visibility mask $B_{vis}$ to the surface consensus to exclude the occluded surface:

(13) $$B_{vis}\left(x,y,d\right)=\{\begin{array}{cc} 1 & SoftVis\left(x,y,d\right)\neq 0\\ 0 & otherwise \end{array}\;.$$

In the right of Figure 5, two images of consensus surfaces are shown. Two images are generated from the consensus volume of the center (reference) from the total of seven input images. The red boxes show some occluded areas from the center view. In the top-right image, the surface consensus is represented without a visibility mask. Therefore, many erroneous background surfaces are shown in the red boxes. In contrast, using the binary mask, the surface consensus represents the only visible surface from the reference view.

**Figure 6:**
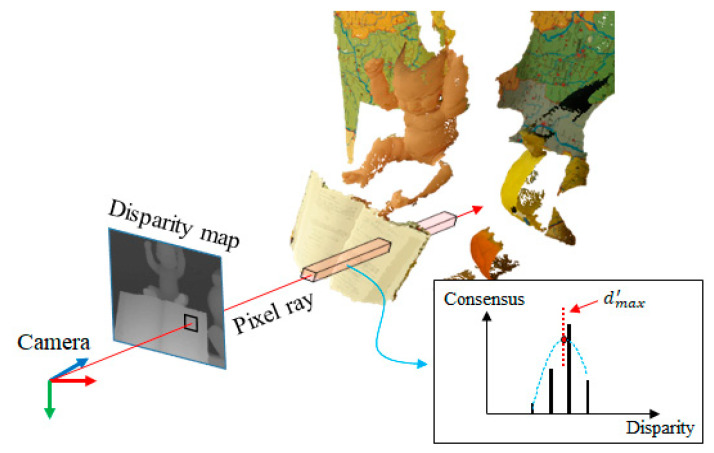
Sub-pixel surface computation in 3D space. In each pixel ray, the maximum consensus is decided and the object surface in sub-pixel accuracy is computed using the quadratic interpolation.

The consensus volume is represented in discontinuous space (due to integer disparity values). In order to use the consensus to efficiently update the PSS matching cost, it is required to represent the surface position (consensus) in the sub-pixel accuracy. To represent the object surface in the sub-pixel accuracy, quadratic interpolation (Quad) is used to find the sub-pixel peak position of the surface, as shown in Figure 6. Given the pixel position in the consensus volume, we first find the peak consensus along the disparity axis ($d_{max}$). Then, the sub-pixel position ($d_{max}^{\prime }$) is interpolated using $d_{max}$ and neighbor voxels as follows:

(14) $$d_{max}\left(x,y\right)=\underset{d\in D}{argmax}(B_{vis}\left(x,y,d\right)\cdot \;Consensus_{r}\left(x,y,d\right))\;,$$

(15) $$d_{max}^{\prime }\left(x,y\right)=Quad\left(d_{max}-1,d_{max},d_{max}+1\right)\;.$$

After all the peak consensus positions are interpolated in the reference image, the PSS matching cost is updated. After several iterations, the sub-pixel surface position $d_{max}^{\prime }$ becomes very accurate. Accordingly, the PSS matching cost is also accurately updated.

### 4.2. Update of Multi-View Matching Cost

The goal of EnSoft3D is to iteratively update the initial PSS multi-view matching costs using the surface consensus. This means that the matching cost of a voxel corresponding to the object surface should be minimized. Because the matching cost is the measure of color error between the matched pixels, the matching cost between the correct pixels must be at a minimum. In the proposed EnSoft3D method, we use the surface consensus to reduce the matching cost at the correct disparity position.

If the initial surface consensus is perfectly computed, it can be used to reduce the matching cost at a correct disparity position. However, the surface consensus can also contain erroneous values and, by reducing only one matching disparity, the local minima problem can occur. Therefore, similar to [7], we use the Gaussian kernel at the consensus peak position to reduce the matching costs of neighbor disparities at the same time. To reduce the PSS matching costs around the consensus peak, an inverse Gaussian kernel is used as shown in (16):

(16) $$V_{r,i}^{new}\left(x,y,d\right)=V_{r,i}\left(x,y,d\right)\;\cdot \left(1-\beta^{\prime }\left(x,y\right)\cdot exp\left(-\frac{{\left(d_{max}^{\prime }\left(x,y\right)-d\right)}^{2}}{2\sigma^{2}}\right)\right).$$

The inverse Gaussian kernel is derived using $d_{max}^{\prime }\left(x,y\right)$ and the standard deviation σ. When the Gaussian value becomes close to 1, $V_{r,i}^{new}\left(x,y,d\right)$ becomes close to zero. Updating the matching cost with too low a value causes another local minimum problem. Therefore, caution must be taken so that the value of $V_{r,i}^{new}\left(x,y,d\right)$ is not too low. Sometimes, the matching cost can be very actively updated in some strong texture areas. Therefore, we employ and multiply an adaptive weight $\beta^{\prime }\left(x,y\right)$ to adjust the Gaussian peak values:

(17) $$var\left(x,y\right)=\frac{1}{n_{w}}\sum_{w}{\left(p\left(x_{w},y_{w}\right)-\frac{\sum_{w}p\left(x_{w},y_{w}\right)}{n_{w}}\right)}^{2},$$

(18) $$var_{n}\left(x,y\right)=\frac{var\left(x,y\right)}{var\left(x,y\right)+\epsilon }\;,$$

(19) $$\beta \left(x,y\right)=\tau_{intensity}\cdot exp\left(\gamma \cdot var_{n}\left(x,y\right)\right)\;,$$

(20) $$\beta^{\prime }\left(x,y\right)=\{\begin{array}{cc} \beta \left(x,y\right) & var_{n}\left(x,y\right)<\tau_{variance}\\ 0.02 & otherwise \end{array},\;$$

where variance var( x,y) is computed within a window (w) and $var_{n}\left(x,y\right)$ is a normalized variance using a parameter ϵ. Adaptive weight is computed by$\;var_{n}\left(x,y\right)$ and a constant γ. The $\tau_{intensity}$ is an update weight to avoid an excessive update, and this weight is used when the variance of a pixel is smaller than threshold $\tau_{variance}$. Other pixels are updated weakly to avoid local minima.

The PSS matching cost volumes are iteratively updated by the proposed scheme in EnSoft3D. The refined matching costs and soft visibility are re-integrated as described in Section 4.3. Since the accuracy of the multi-view matching cost is improved, it is also possible to obtain a more accurate disparity map than Soft3D. With this proposed update scheme, both the consensus and soft visibility become more accurate in the next iteration, and the improved properties further improve the final disparity map generation.

**Figure 7:**
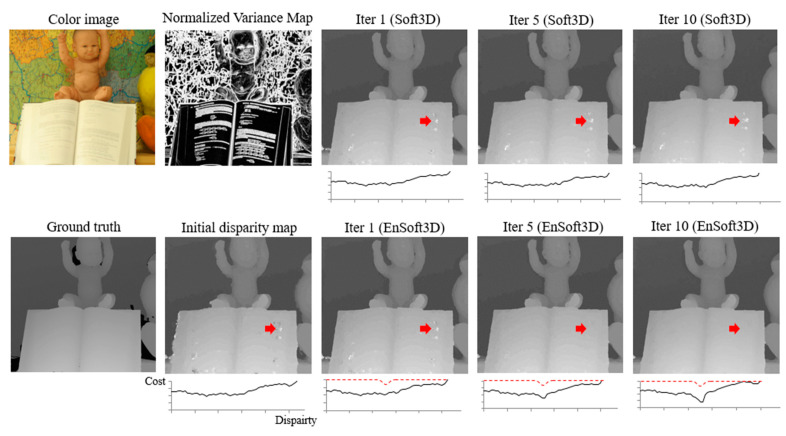
Visualization of the refined disparity map and PSS matching cost in the ray at the red arrow pixel (Baby2). It shows that the proposed method correctly updates the matching cost by an inverse Gaussian kernel (red-dotted line) computed by using object surface consensus.

A comparison of the matching cost update between Soft3D and EnSoft3D is shown in Figure 7. The disparity image in the graph below shows the matching costs of a pixel at the red arrow in the figure. In Soft3D, the matching cost at the incorrect disparity position does not change because the PSS matching cost is not refined. In contrast, the matching cost of EnSoft3D is iteratively updated and reaches a minimum at the correct disparity. As shown in the disparity maps, it is known that the proposed method can improve the performance of the disparity generation more than Soft3D. 

### 4.3. New Matching Cost Integration

The Soft3D method generates a new cost volume $V_{r}\prime \left(x,y,d\right)$ which stores the result of the weighted average of soft visibility and PSS matching costs, as in (12). In contrast, in EnSoft3D, the results of the weight average are stored at the initial cost volume $V_{r}\left(x,y,d\right)$ which is defined in (6). The proposed computation of $V_{r}\left(x,y,d\right)$ using the updated PSS matching costs is shown in (21). The reason for a different method of storage is that the 3D reconstruction requires an estimation of the disparity value at a sub-pixel accuracy. For sub-pixel disparity estimation, we perform quadratic interpolation as the last step in the 3D reconstruction. This step uses $V_{r}\left(x,y,d\right)$ to interpolate d and its neighbors, d−1 and d+1 for sub-pixel disparity in each pixel ray. However, if the soft visibility of all corresponding disparities is 0 (such as behind the object surface), $V_{r}\left(x,y,d\right)$ cannot be integrated. In this case, if the integrated cost is stored in a new volume, $V_{r}\prime \left(x,y,d\right)$, some pixels cannot be interpolated due to empty visibility voxels. To prevent this kind of interpolation error, we store the integrated costs in $V_{r}\left(x,y,d\right)$. With this new integration method, only the visible voxels of $V_{r}\left(x,y,d\right)$ are updated, and non-updated voxels of $V_{r}\left(x,y,d\right)$ keep the previous values. 

After $V_{r}\left(x,y,d\right)$ is computed, the guided filter is applied to every inverse depth plane and a refined disparity map is computed by referring only to the updated voxel (processing only visible areas) with the following equation:

(21) $$V_{r}\left(x,y,d\right)=\frac{\sum_{i\in N-1}V_{r,i}^{new}\left(x,y,d\right)SoftVis_{i}\left(x_{i}^{\prime },y_{i}^{\prime },d_{i}^{\prime }\right)}{\sum_{i\in N-1}SoftVis_{i}\left(x_{i}^{\prime },y_{i}^{\prime },d_{i}^{\prime }\right)}\;,$$

(22) $$D_{r}^{new}\left(x,y\right)=argmin_{d\in D}(\left(1-B_{r}\left(x,y,d\right)\;\cdot \tau_{max}+B_{r}\left(x,y,d\right)\cdot V_{r}\left(x,y,d\right)\right)\;,$$

where$\;B_{r}\left(x,y,d\right)$ is a binary flag indicating whether the voxel (x,y,d) is updated or not, and $\tau_{max}$ is a threshold value for avoiding the non-updated voxel.

The refined disparity map $D_{r}^{new}$ is again used as an input to the EnSoft3D multi-view stereo matching scheme. After a sufficient number of iterations, both the soft visibility and PSS matching costs are correctly refined, and very accurate disparity maps are obtained.

## 5. Experiment and Evaluation

In this section, the performance of proposed method is compared with the state-of-the-art multi-view stereo matching and view synthesis methods. For the experiments, we use two types of multi-view stereo images. One consists of structured multi-view images obtained with a calibrated array camera and the other consists of unstructured multi-view images obtained with a single moving camera. If the camera’s intrinsic and pose parameters are not available, we use the COLMAP [32] algorithm for calibration. 

The first evaluation is completed with a structured multi-view image dataset *Middlebury* which is famous for stereo vision. The 3D reconstruction performance is measured by the ratio of bad pixels. The view synthesis evaluation is measured by the quality of synthesized images. The view synthesis is completed with the algorithm in Soft3D. For the quantitative analysis of view synthesis, we measure the peak signal-to-noise ratio (PSNR) and structural similarity index map (SSIM) between the synthesized image and the ground truth image. 

The second evaluation is completed by using the unstructured multi-view image dataset. In order to show the effect of the proposed matching cost refinement, we use multi-view images which have a small camera movement so that all the images have common overlapping areas. *Fountain-P11* and part of the *ETH low-resolution* dataset are used as the unstructured multi-view image dataset.

Our computing environment is as follows: Intel i9-10900K, 64GB RAM, and sources which are implemented by C++. The number of inverse depth planes in the PSS algorithm is between 80 and 120. The refinement iteration is completed a minimum of five to a maximum of ten times.

### 5.1. Structured Dataset: Middlebury

#### 5.1.1. D Reconstruction of Middlebury 2003 Dataset

**Figure 8:**
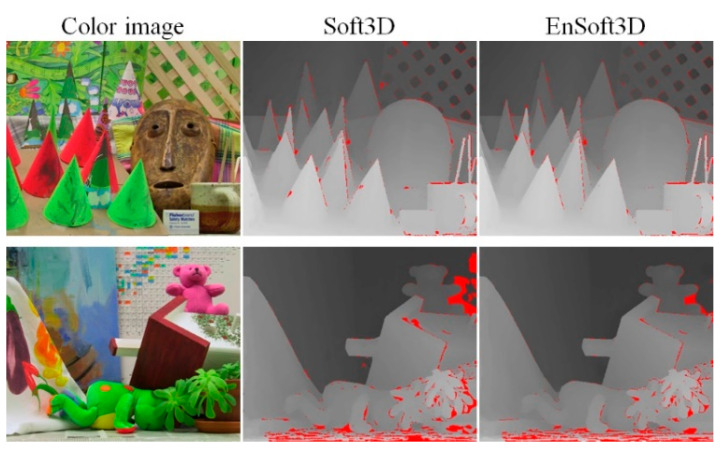
Comparison of Soft3D and EnSoft3D using *Middlebury 2003* dataset (*Cones, Teddy*). The red color areas in disparity map are bad pixels when the error threshold is 1.0.

*Cones* and *Teddy* are famous images in the *Middlebury 2003* dataset for stereo matching evaluation. In our evaluation, we use nine-view quarter-size images. A visual comparison between EnSoft3D and Soft3D is shown in Figure 8. The red-colored pixels in the disparity map are bad pixels when the error threshold is one pixel. Compared with Soft3D, EnSoft3D shows a smaller number of bad pixels. In the Teddy image, some pixels consist of a repeated pattern and a large textureless area. In this area, EnSoft3D cannot sufficiently refine the depth due to the incorrect surface consensus.

For a detailed quantitative analysis, our results are compared with many stereo matching algorithms evaluated in the *Middlebury v2* benchmark [33], as shown in Table 1. Among a total of 167 algorithms, 20 well-known and high-ranking algorithms of average accuracy are compared. In addition, a couple of multi-view stereo algorithms, MVSegBP and MultiCamGC, are also compared. In this comparison, three different measurements are used for bad pixels; nonocc, all, and disc mask. The nonocc evaluates the pixels excluding the occlusion area, the all evaluates all pixels, and the disc evaluates only the discontinuous area. In each table, 22 algorithms including EnSoft3D and Soft3D are ranked by the bad pixel ratio when the error threshold is 0.5 pixels for the all mask. In the *Cones* image, EnSoft3D ranks 1st Top one and the bad pixel ratio is 5.35. When the error threshold is 1.0, EnSoft3D ranks 2nd behind the PM-Forest [34] and the bad pixel ratio is only 2.73. In *Teddy* image, our method ranks 4th when the threshold is 0.5 pixel and 2nd when the threshold is 1.0 pixels.

The performance of the proposed method depends on the accuracy of consensus and visibility. For example, if the region of the textureless or repeated pattern is wide, both the consensus and visibility can be incorrectly computed due to color ambiguity. In this reason, the effect of iterative enhancement of PSS cost can be reduced. After several real experiments, we find that some pixels in such textureless regions can be refined after a large number of iterations. However, this requires a long processing time and can cause the local minimum problem in PSS cost update. This is why almost all bad pixels in *Teddy* are distributed at the bottom of the image, a textureless planar region as shown in Figure 8. This problem could be improved using an additional process in consensus computation, such as planar surface fitting.

#### 5.1.2. D Reconstruction of Middlebury 2006 Dataset

As the second evaluation for 3D reconstruction, we use the *Middlebury 2006* dataset to compare the disparity error of the proposed method with a variety of two-view and multi-view stereo matching methods. For a fair comparison, we refer to two recent stereo matching papers [35,36] for a quantitative error analysis of the disparity image reconstruction. In [35], a multi-scale fusion stereo matching method was proposed to obtain an accurate disparity in the textured and textureless regions. Table 6 in [35] presents and compares the disparity errors of eight different methods, including the author’s method, using the *Middlebury 2006* dataset. In this comparison, several patch-match-based methods are also included. In [36], a matching cost volume filtering method named FASW is proposed. Table 5 in [36] also presents the disparity errors of five different methods using the same *Middlebury 2006* dataset. 

We tested Soft3D and EnSoft3D using 17 images among the images in the two tables in [35,36]. In [35], a total of twenty-one images are compared. Among them, four images, *Midd1*, *Midd2*, *Monopoly* and *Plastic,* are excluded from our comparison because those images are composed of very large textureless regions and our method does not specifically address the stereo matching of textureless objects. However, images such as *Lampshade1*, *Lampshade2*, *Bowling1*, and *Bowling2*, which are composed of moderately textureless regions, are included in our comparison. In [36], a total of twenty-seven images are compared. Among them, we use 17 images which are common with the test images in [35]. 

**Figure 9:**
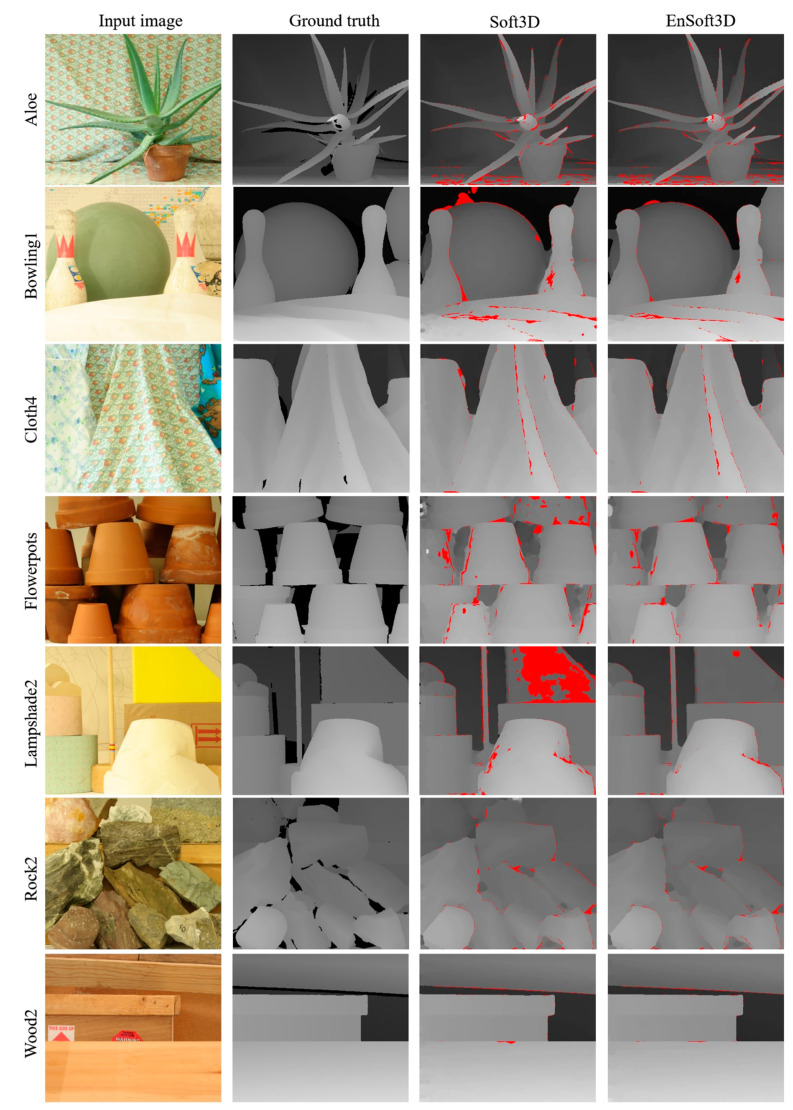
Comparison of Soft3D and EnSoft3D using *Middlebury 2006* dataset. The red-colored areas in disparity map are bad pixels when the error threshold is 1.0 (**nonocc** mask).

The two above papers provide extensive comparison results of recent stereo matching methods using many images from the *Middlebury 2006* images. Therefore, the quantitative comparison of a total of 14 methods listed in the two reference papers can be considered as an objective error analysis of the proposed method. In addition, for fair comparison, we use the same error measurement criteria with the two papers: the error threshold is 1.0 pixel with the **nonocc** mask. Our proposed method shows the best performance in nine images and a good performance in the remaining test images than the other methods including the original Soft3D as shown in Table 2. In terms of the average error of all 17 images, the proposed method is 1.54 pixels and is the best among all methods. Figure 9 shows examples of the final disparity images of seven test images reconstructed by the proposed method. Because not all the disparity images are available in [35,36], we compare our results with the ground truth and Soft3D.

#### 5.1.3. View Synthesis of Middlebury 2006 Dataset

**Figure 10:**
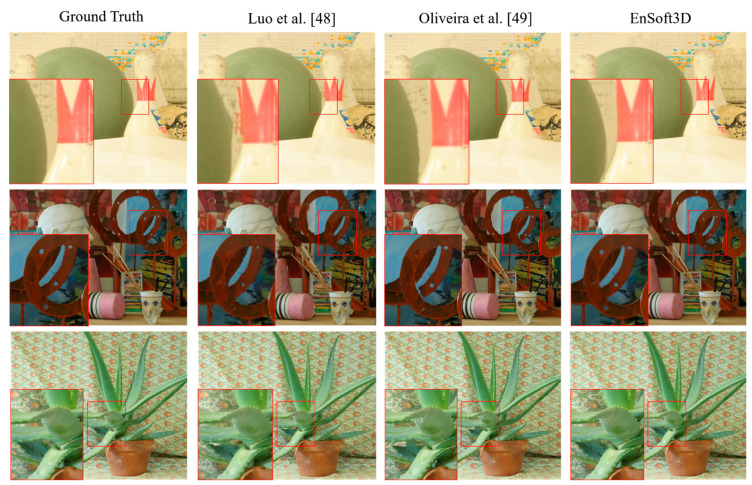
View synthesis result (*Middlebury 2006*: From top, *Bowling1, Art*, and *Aloe*). The red box shows the close-up results. Our results shows high quality images and more close to the ground truth.

The *Middlebury 2006* dataset is also used for view synthesis analysis. *Art, Laundry, Cloth1, Dolls, Books, Wood1, Bowling1, Bowling2, Aloe* are 7-view images and depth maps are generated in six views, with the exception of a test view. The depth of six views are used to synthesize the new image at the test view. In Figure 10, the view synthesis results of *Bowling1, Art*, and *Aloe* are compared with the Luo et al. [48] and Oliveira et al. [49] algorithms. Our results show high quality results in the object edge and corner areas. In the close-up image in Figure 10, we find that the object edge of our result is synthesized in a very similar way to the original color image. The PSNR and SSIM measurements of all nine test images are shown in Table 3. For a sufficient number of comparisons, we use six algorithms, including Jain et al. [50], Tran et al. [51], Ramachandran et al. [52] and Soft3D. In our results, the PSNR ranges from 38 to 45 and the SSIM ranges from 0.97 to 0.99, which are the highest scores among all the compared methods. Compared to Soft3D, a view synthesis using the refined depth shows a better performance. This implies that the PSS matching costs are updated correctly by the surface consensus.

### 5.2. Unstructured Dataset: Fountain-P11, ETH3D Low Resolution Dataset

Another evaluation is completed using unstructured multi-view images. In Section 5.1, the structured multi-view images are captured by a camera moving in the horizontal direction, which results in an x-axis image shift only. In the unstructured multi-view images, the camera moves in a free space and the 6-DoF external parameters are computed for multi-view matching. In this experiment, we compare the 3D reconstruction performance with state-of-the-art algorithms. 

The first dataset is *Fountain-P11*. These data consist of 11 images, where the camera motion is large. We reconstruct the 3D point cloud using 768 × 512 resolution images. In the unstructured real scene data, especially in the outdoor environment, the matching cost computation is difficult due to the large depth range between the foreground and background objects. To enhance the depth resolution, we increase the number of inverse depth planes. Increasing depth resolution produces more accurate 3D point clouds. However, since the cost volume size increases, the processing time also increases.

**Figure 11:**
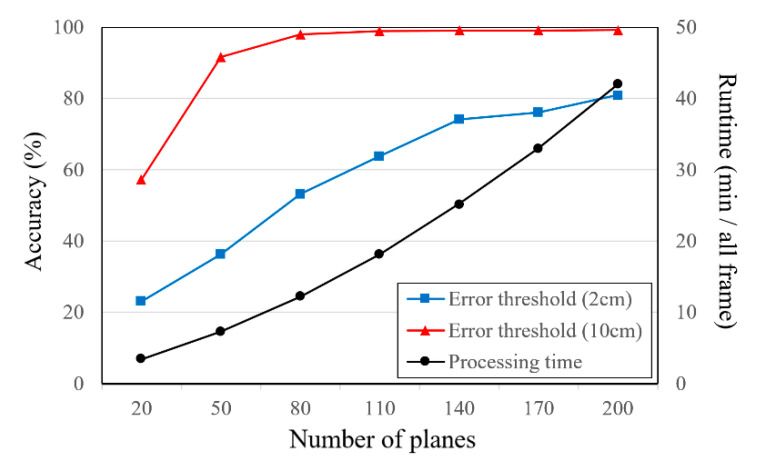
Relationship of accuracy (ratio of pixels) and runtime with respect to the number of inverse depth planes (*Fountain-P11*).

The relationship between the accuracy and processing time with respect to the number of inverse depth planes is shown in Figure 11. In this experiment, the 3D point cloud is reconstructed using 11 views (768 × 512 pixels) and 10 iterations of refinement. When the error threshold is 2 cm, the accuracy improves as the number of planes increases. Additionally, the processing time increases significantly with the number of planes, proportionately. In contrast, when the error threshold is 10 cm, the accuracy of more than 80 planes is saturated.

**Figure 12:**
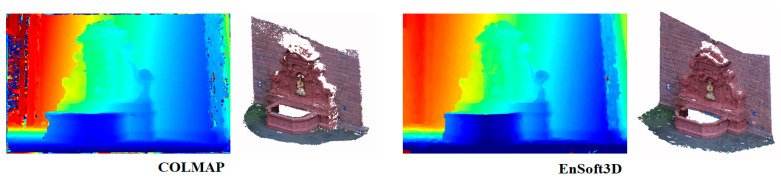
Comparison of 3D reconstruction performance with COLMAP (quality set: extreme). The depth map is the result of view-2, and the point clouds are fused from all views.

Based on this experiment, we use 80 to 120 disparity planes in the indoor environment, and more in the outdoor environment. Figure 12 shows the result of the 3D reconstruction of the second view of *Fountain-P11*. Our method reconstructs more dense point clouds compared with the same input resolution in COLMAP. It is shown that the speckle noises are very low in the object edge area. The quantitative analysis of *Fountain-P11* is shown in Table 4. In this table, the ratio of the correct pixels with respect to the error threshold is shown. When the ratio is closer to 1, the performance is better. Analysis of many other algorithms are cited from the COLMAP paper. When the error threshold is 10 cm, our result with more than 80 planes is better than the other algorithms.

**Figure 13:**
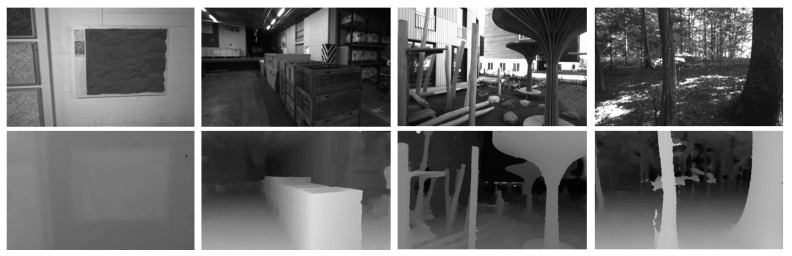
Our result using the *ETH3D low-resolution* dataset (*Terrains, Delivery area, Playground, Forest*). These results are generated using only 16 views.

**Figure 14:**
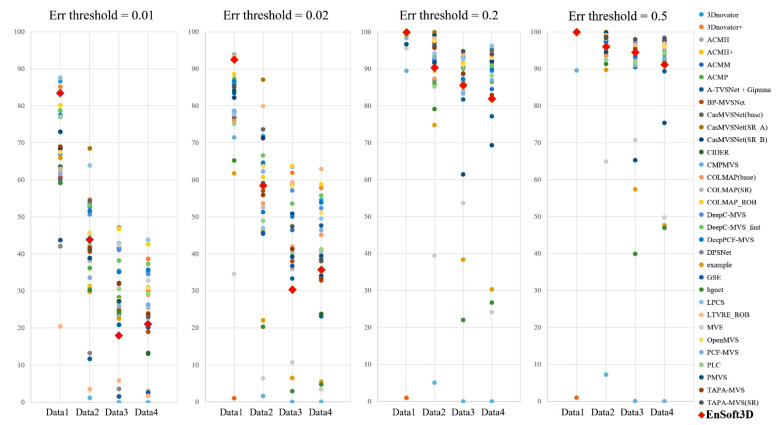
*ETH3D* online benchmark (online: [60]) accuracy comparison using low-resolution dataset (Data 1-4 is *Terrains, Forest, Playground, Delivery area*). From the left, each graph shows the ratio of pixels where error is less than 0.01, 0.02, 0.2, and 0.5 m, respectively.

The second unstructured multi-view is the *ETH3D low-resolution* dataset. These data are obtained by four cameras, and each camera consists of 200 to 300 grayscale images. Because the proposed algorithm has a limitation in the number of input images, we use 16 images from the first camera. The image resolution is 910 × 510 pixels. We evaluate the accuracy of the 3D point clouds and compare this with the *ETH3D* online benchmark [60], as shown in Figure 13 and Figure 14. In this benchmark test, the accuracies of three deep learning methods are also compared (DeepC-MVS, DeepPCF-MVS, and DPSNet, explained in Section 2). Recently, deep learning methods show a competitive performance in the multi-view stereo benchmark test. In multi-view stereo matching, the depth accuracy is improved when many images are used. Most algorithms in the *ETH3D* online benchmark use all of the input images. However, our method uses only 16 images, which cannot be a fair comparison. Nevertheless, the proposed method can obtain a dense depth even with a small number of images. Using an indoor dataset, *Terrains*, 3D reconstruction can be completed in high quality (the accuracy in a 2 cm error threshold is 92.55%). In this comparison, the accuracies of DeepC-MVS, DeepPCF-MVS, and DPSNet are 86.38%, 86.72%, and 65.18%, respectively. In contrast, a reconstruction using an outdoor dataset shows an inaccurate performance. For example, in *Forest*, the accuracy of the proposed method is 58.52% when the error threshold is 2 cm. This is a better accuracy than DPSNet (20.25%); however, it is lower than DeepC-MVS (63.91%) and DeepPCF-MVS (64.65%). This is a problem with the disparity space in the PSS algorithm. The PSS algorithm is completed in the inverse depth planes which are defined by a constant disparity interval. This causes a large depth interval in the far distance of the real metric space. For this reason, the depth interpolation with the sub-pixel accuracy is relatively inaccurate in the far distance, which results in a low depth accuracy in 3D reconstruction.

As an additional experiment, the 3D reconstruction accuracy is measured within 7 m. This experiment is to show the better performance of the proposed method in the near distance. The error thresholds are set to 0.1, 0.2, and 0.5 m. The results of the *Delivery area* are 88.23%, 94.34%, and 97.78%, respectively. The results of *Playground* are 85.71%, 93.74%, and 97.32%, and *Forest* is 93.55%, 96.79%, and 98.73%. From this experiment, we find that accurate 3D reconstruction is achievable using the dense depth interval in the PSS algorithm.

### 5.3. Runtime

In this section, the runtime of the proposed method is analyzed and compared with different methods. To compare the runtime with other methods, we refer to Table 3 in [36] and Table 8 in [61] which use the *Middlebury* dataset as test images. In Table 3 of [36], the processing times of 11 methods are presented with the computation environments of each method. The stereo matching runtime is measured with the quad resolution image pairs of the *Middlebury* dataset. In Table 8 of [61], the runtime of stereo matching with three different image resolutions is compared. In this table, the quad, half, and original resolutions of the *Middlebury* dataset are compared with an Intel i7 CPU with a GeForce GTX 1080 Ti GPU. 

We cite the two tables in [36,61] to compare the runtime of the proposed method. In Table 5 below, the runtime of Soft3D and EnSoft3D are added to Table 3 in [36]. The processing is the measured time for generating a single frame of the depth image. Additionally, the computation environment is an Intel i9-10900K CPU with 64GB RAM. In Table 6, three different resolutions of images are used to compare the runtime of the proposed method, quad, half, and full resolutions of the Middlebury dataset. The runtime of the proposed method is reasonably fast compared with three different methods. 

As shown in the two tables, there is no conspicuous time difference between the Soft3D and EnSoft3D methods. Because the update process of the PSS cost can be considered as the 2D convolution of a Gaussian-like kernel, it only needs an additional short processing time. 

### 5.4. Limitations

In this section, we discuss some limitations of the proposed EnSoft3D method. The first limitation is memory usage. All processes in the proposed method require a lot of 3D memory space. Each view must have multiple matching costs, consensuses, and visibility volumes. The 3D volume size is determined by the resolution of the input image and the disparity range (width × height × disparity). Therefore, with a large number of views or image resolutions, the proposed method cannot be executed due to memory limitations. In addition, the problem of the sub-pixel accuracy described in Section 5.2 is also related to memory limitation. In the PSS algorithm, the larger the interval between the inverse depth planes, the less accurate the 3D reconstruction is. Increasing the number of planes improves the accuracy; however, it requires more memory.

Using many 3D volumes also requires a long processing time. Multi-view matching, soft visibility computation, and cost updates are performed simultaneously in all multi-view images. In our computation environment, it takes 2 min and 40 s (initial disparity computation: 20 s, refinement of 5 iterations: 2 min 20 s) to produce half-size seven-views disparity maps in the *Middlebury* dataset. To reduce the processing time, GPU implementation using a large video memory must be used.

The last limitation is the refinement of the repeated image pattern and large textureless area. Even in this area, the proposed method can refine the matching costs when a large number of iterations is used. However, the results are not perfect. Because the erroneous surface consensus is widely distributed in this area, the edge-preserving filter cannot remove the errors. To solve this problem, an additional refinement method, such as depth plane fitting, is needed.

## 6. Conclusions

In this paper, we propose EnSoft3D, a multi-view stereo matching method for high-quality 3D reconstruction and view synthesis. In contrast to our base algorithm, Soft3D, we introduce a new multi-view stereo matching scheme for the generation of dense and accurate disparity maps. Multi-view stereo matching costs and a soft visibility are simultaneously updated by using the surface consensus in the 3D inverse depth space. 

The first step of the proposed method is to decide the position of the object surface in the disparity space. We use the consensus volume. This is the likelihood of the distribution of the object surface in the disparity space. Based on this likelihood, we derive the surface consensus in the sub-pixel accuracy by quadratic interpolation. The second step is the matching cost update process. An inverse Gaussian kernel is generated based on the surface consensus. By multiplying the inverse Gaussian kernel, the matching costs corresponding to the surface voxels are reduced. The level of reduction is adjusted by the color variance of pixels. This update process is repeated simultaneously with the soft visibility update process. 

The quantitative analysis of the proposed method is completed by using the structured and unstructured multi-view stereo datasets, *Middlebury 2003, 2006, Fountain-P11,* and *ETH3D low-resolution datasets*. We completed extensive error analyses and comparisons, including a disparity estimation, a view synthesis of image quality, and a 3D reconstruction performance. In addition, the runtime was analyzed and compared with other methods. As shown in the experimental results, the proposed method achieved the best performance in both 3D reconstruction and view synthesis.

## Figures and Tables

**Table 1:** The ratio of bad pixels when error threshold is 0.5 and 1.0 pixel. Evaluation and ranking are from the *Middlebury v2* benchmark (online: [33]) (*Cones, Teddy*). The ranking of listed algorithms is sorted in the descending order when error threshold is 0.5 for all pixels.

***Cones* (# of Disparity Plane: 80)**	**Err Threshold: 0.5**			**Err Threshold: 1.0**		
**Algorithm (Online rank)**	**nonocc**	**all**	**disc**	**nonocc**	**all**	**disc**
**EnSoft3D**	**2.85**	**5.35**	**7.41**	**1.42**	**2.73**	**4.22**
PM-Forest (1)	5.11	6.35	9.31	1.32	2.02	3.69
**Soft3D**	**3.66**	**6.75**	**9.13**	**1.63**	**3.35**	**4.78**
PM-Huber (2)	2.70	7.90	7.77	2.15	6.69	6.4
ARAP (4)	3.00	8.55	8.35	2.08	6.73	6.17
GC+LocalExp (6)	3.46	8.65	9.72	2.72	7.42	7.94
PM-PM (7)	3.51	8.86	9.58	2.18	6.43	6.37
SubPixSearch (10)	4.02	9.76	10.3	2.24	6.87	6.5
PatchMatch (11)	3.80	10.2	10.2	2.47	7.80	7.11
LAMC-DSM (17)	4.00	11.0	9.79	2.09	8.31	6.1
C-SemiGlob (23)	5.37	11.7	12.8	2.77	8.35	8.2
IGSM (25)	6.17	11.8	12.0	2.14	6.97	6.27
ADCensus (28)	6.58	12.4	11.9	2.42	7.25	6.95
SemiGlob (30)	4.93	12.5	13.5	3.06	9.75	8.9
CrossLMF (46)	7.55	13.5	14.5	2.34	7.82	6.8
BSM (56)	6.45	14.1	13.1	2.34	8.79	6.8
SubPixDoubleBP (70)	8.49	14.7	16.5	2.93	8.73	7.91
AdaptLocalSeg (76)	7.78	15.0	16.2	2.73	9.69	7.91
HistAggrSlant (81)	9.34	15.1	16.2	2.90	8.40	7.97
CVW-RM (106)	12.6	17.9	18.6	2.96	7.71	7.72
MultiCamGC (113)	12.0	18.8	21.2	4.89	11.8	12.1
MVSegBP (130)	14.4	21.2	24.5	5.29	11.3	14.5
***Teddy* (# of disparity plane: 200)**	**Err threshold: 0.5**			**Err threshold: 1.0**		
**Algorithm (Online rank)**	**nonocc**	**all**	**disc**	**nonocc**	**all**	**disc**
PM-Forest (1)	4.95	5.45	11.3	1.91	2.29	5.47
GC+LocalExp (3)	5.16	7.73	14.2	3.33	4.88	8.87
PM-Huber (5)	5.53	9.36	15.9	3.38	5.56	10.7
**EnSoft3D**	**6.77**	**9.94**	**17.4**	**3.86**	**4.85**	**10.9**
ARAP(8)	5.52	10.7	15.6	3.01	6.47	9.51
SubPixSearch (9)	6.71	11.0	16.9	4.00	6.39	11.0
**Soft3D**	**9.61**	**11.3**	**21.4**	**5.91**	**6.57**	**13.8**
PatchMatch (10)	5.66	11.8	16.5	2.99	8.16	9.62
PM-PM (11)	5.21	11.9	15.9	3.00	8.27	9.88
IGSM (13)	9.02	12.1	21.3	4.08	5.98	11.4
ADCensus (15)	10.6	13.8	20.1	4.1	6.22	10.9
LAMC-DSM (19)	7.29	14.6	19	4.63	10.4	12.7
BSM (21)	11.2	15.2	24.2	5.74	8.95	14.8
SubPixDoubleBP (26)	10.1	16.4	21.3	3.45	8.38	10.0
C-SemiGlob (36)	9.82	17.4	22.8	5.14	11.8	13.0
CrossLMF (37)	11.1	17.5	24.1	5.50	10.6	14.2
HistAggrSlant (40)	11.2	17.6	22.5	3.44	8.82	9.77
CVW-RM (48)	15.0	17.9	26.2	4.70	6.94	12.1
SemiGlob (56)	11.0	18.5	26.1	6.02	12.2	16.3
AdaptLocalSeg (80)	12.9	20.0	26.5	5.32	11.9	14.5
MVSegBP (93)	15.9	21.5	29.8	6.53	11.3	14.8
MultiCamGC (153)	24.3	30.4	36.9	12.0	17.6	22.0

**Table 2:** The ratio of bad pixels from *Middlebury 2006* dataset (error threshold: 1.0 pixel, **nonocc** mask).

Dataset	[37]	[38]	[39]	[40]	[41]	[42]	[43]	[35]	[44]	[45]	[46]	[47]	[36]	Soft3D	EnSoft3D
*Aloe*	4.51	6.75	3.21	5.03	3.92	3.78	3.06	4.15	5.17	6.19	6.51	6.94	4.50	3.32	**2.98**
*Baby1*	4.10	3.27	2.21	4.45	2.74	2.15	1.98	**1.40**	3.01	7.37	3.23	3.19	2.26	1.57	1.49
*Baby2*	4.77	3.97	2.08	16.9	5.48	2.87	**1.05**	1.49	3.60	13.96	3.77	4.21	3.51	1.55	1.38
*Baby3*	4.77	3.92	3.07	4.54	6.56	3.22	3.12	2.38	4.31	7.85	4.63	4.77	3.76	1.85	**1.74**
*Bowling1*	14.1	12.1	4.14	16.5	5.37	5.30	2.06	3.92	7.58	17.17	5.71	6.38	5.76	3.29	**1.56**
*Bowling2*	4.64	5.27	2.19	10.6	6.44	2.74	**1.45**	2.64	7.49	12.58	7.81	7.40	5.29	3.53	3.34
*Cloth1*	1.68	1.17	0.71	0.68	0.78	0.89	0.60	0.71	0.77	0.96	1.65	1.09	0.66	0.41	**0.34**
*Cloth2*	4.19	4.52	2.90	4.30	3.26	2.81	2.39	2.40	2.80	4.60	3.82	3.28	2.15	1.47	**1.38**
*Cloth3*	2.88	2.15	1.66	2.54	1.62	1.91	1.57	1.01	2.06	2.55	2.48	2.73	1.68	0.99	**0.94**
*Cloth4*	3.10	2.20	1.75	1.70	0.99	1.78	1.87	**0.79**	1.74	1.86	2.00	2.06	1.31	1.08	0.86
*Flowerpots*	9.28	8.80	4.60	12.5	10.8	5.56	2.49	**1.81**	9.32	15.51	8.87	9.71	8.97	4.87	2.59
*Lampshade1*	13.5	8.67	12.6	10.9	5.96	9.70	**1.50**	1.63	9.85	10.96	9.91	14.80	6.37	10.5	1.95
*Lampshade2*	16.5	17.2	10.0	16.2	21.7	11.1	**0.99**	1.76	9.52	12.69	10.65	16.93	6.42	13.9	1.27
*Rock1*	4.15	2.60	2.19	2.96	1.60	2.35	1.80	1.28	2.52	3.63	3.61	4.06	2.22	1.16	**1.04**
*Rock2*	1.82	1.68	1.46	2.38	1.49	1.64	1.20	0.94	2.00	2.91	2.50	2.76	1.75	1.02	**0.89**
*Wood1*	1.52	4.19	0.48	4.66	1.30	0.87	**0.73**	3.36	5.16	10.53	4.55	4.95	2.95	2.16	2.14
*Wood2*	0.70	0.49	0.36	2.38	4.62	0.56	0.37	0.42	3.43	5.95	2.75	2.53	2.42	0.45	**0.36**
**Average**	5.66	5.23	3.27	7.01	4.98	3.48	1.66	1.89	4.73	8.07	4.97	5.75	3.65	3.12	**1.54**

**Table 3:** PSNR and SSIM view synthesis evaluation using the *Middlebury 2006* dataset.

Middlebury Dataset	[50]		[51]		[52]		[48]		[49]		Soft3D		EnSoft3D	
	PSNR	SSIM	PSNR	SSIM	PSNR	SSIM	PSNR	SSIM	PSNR	SSIM	PSNR	SSIM	PSNR	SSIM
*Art*	31.67	0.95	32.09	0.94	30.22	0.94	27.85	0.91	30.02	0.93	38.71	0.97	**39.25**	**0.97**
*Laundry*	31.66	0.95	31.63	0.95	31.32	0.94	28.85	0.93	29.99	0.94	38.33	0.97	**38.59**	**0.98**
*Cloth1*	35.04	0.96	35.68	0.97	33.66	0.94	-	-	-	-	43.39	0.99	**43.43**	**0.99**
*Dolls*	31.61	0.95	31.95	0.94	30.90	0.94	-	-	-	-	41.96	0.98	**42.02**	**0.98**
*Books*	30.10	0.93	30.15	0.92	38.74	0.92	-	-	-	-	41.12	0.98	**41.22**	**0.98**
*Wood1*	36.29	0.94	37.36	0.94	-	-	-	-	-	-	44.71	0.98	**44.75**	**0.99**
*Bowling1*	-	-	-	-	-	-	31.97	0.95	32.23	0.95	42.33	0.98	**45.08**	**0.99**
*Bowling2*	-	-	-	-	-	-	28.44	0.93	30.48	0.94	43.35	0.98	**43.43**	**0.98**
*Aloe*	-	-	-	-	-	-	29.91	0.92	29.35	0.92	38.75	0.97	**38.89**	**0.98**

**Table 4:** Ratio of pixels where error is less than 2 and 10cm in *Fountain-P11*. The P80, P140, and P200 are results when the number of inverse depth planes in the PSS algorithm is 80, 140, and 200.

		[53]	[54]	[55]	[56]	[57]	[58]	[59]	[32]	Soft3D			EnSoft3D		
										(P80)	(P140)	(P200)	(P80)	(P140)	(P200)
Fountain	**2 cm**	0.769	0.754	0.731	0.712	0.732	0.824	0.693	**0.827**	0.496	0.711	0.789	0.532	0.741	0.810
	**10 cm**	0.929	0.930	0.838	0.832	0.822	0.973	0.838	0.975	0.964	0.983	0.987	0.980	0.991	**0.993**

**Table 5:** Runtime comparison of *Middlebury* quad resolution dataset.

Algorithm	Environments	Runtime (s)
PSMNet_ROB [62]	Nvidia GeForce GTX 1080 Ti /PCIe /SSE2 (CUDA, Python/PyTorch)	0.55
DSGCA [63]	i7-4770 @ 3.40 GHz; GTX 1080 GPU (Matlab)	11.0
ADSM [64]	8 i7 cores; Nvidia GTX460 SE (CUDA, C/C++)	35.8
DDL [65]	4 i7 cores @ 4.0 GHz (Matlab/C)	112
IGF [66]	1 i5 Core @ 3.2 GHz (C++/OpenCV)	132
BSM [67]	Intel(R) Core(TM)2 Duo CPU P7370 @ 2.00GHz (C++/OpenCV)	244
ISM [47]	1 i5 core @ 3.2 GHz (C/C++)	330
MPSV [68]	1 i5 core @ 2.7 GHz (Python)	594
DogGuided [69]	2 i5 cores @ 3.0 GHz (Matlab)	630
DF [70]	Matlab 2017	9999
FASW [36]	Intel Core i5-6500 @ 3.2 GHz	40.5
Soft3D	Intel Core i9-10900K @ 3.7 GHz (C++/OpenCV)	13.3
EnSoft3D	Intel Core i9-10900K @ 3.7 GHz (C++/OpenCV)	14.1

**Table 6:** Runtime comparison of multi-frame stereo matching methods.

Resolution of Middlebury Dataset	Runtime (Seconds)				
	[71]	[72]	[61]	Soft3D	EnSoft3D
Quad resolution (100–200 kP)	8.2	16.4	10.1	12.1	13.3
Half resolution (200–500 kP)	68.1	139.2	35.8	33.0	34.4
Original resolution (1–2 MP)	458.7	1905.4	170.1	136.1	141.5
Environments	Intel i7	Intel i7+ GTX 1080 Ti	Intel i7	Intel i9	Intel i9

## Data Availability

Not applicable.

## References

[1] Hirschmuller H. Accurate and efficient stereo processing by semi-global matching and mutual information. Proceedings of the IEEE Computer Society Conference on Computer Vision and Pattern Recognition (CVPR).

[2] Yao Y., Luo Z., Li S., Shen T., Fang T., Quan L. Recurrent mvsnet for high-resolution multi-view stereo depth inference. Proceedings of the IEEE/CVF Conference on Computer Vision and Pattern Recognition (CVPR).

[3] Zhang H.T., Yu J., Wang Z.F. (2018). Probability contour guided depth map inpainting and superresolution using non-local total generalized variation. Multimed. Tools Appl..

[4] Penner E., Zhang L. (2017). Soft 3d reconstruction for view synthesis. ACM Trans. Graph..

[5] Collins R.T. A space-sweep approach to true multi-image matching. Proceedings of the IEEE Computer Society Conference on Computer Vision and Pattern Recognition (CVPR).

[6] Ha H., Im S., Park J., Jeon H.G., Kweon I.S. High-quality depth from uncalibrated small motion clip. Proceedings of the IEEE Conference on Computer Vision and Pattern Recognition (CVPR).

[7] Poggi M., Pallotti D., Tosi F., Mattoccia S. Guided stereo matching. Proceedings of the IEEE/CVF Conference on Computer Vision and Pattern Recognition (CVPR).

[8] Scharstein D., Szeliski R. High-accuracy stereo depth maps using structured light. Proceedings of the IEEE Computer Society Conference on Computer Vision and Pattern Recognition (CVPR).

[9] Hirschmuller H., Scharstein D. Evaluation of cost functions for stereo matching. Proceedings of the IEEE Conference on Computer Vision and Pattern Recognition (CVPR).

[10] Schops T., Schonberger J.L., Galliani S., Sattler T., Schindler K., Pollefeys M., Geiger A. A multi-view stereo benchmark with high-resolution images and multi-camera videos. Proceedings of the IEEE/CVF Conference on Computer Vision and Pattern Recognition, (CVPR).

[11] Strecha C., Hansen W.V., Gool L.V., Fua P., Thoennessen U. On benchmarking camera calibration and multi-view stereo for high resolution imagery. Proceedings of the IEEE Conference on Computer Vision and Pattern Recognition (CVPR).

[12] Huynh T.Q., Ghanbari M. (2012). The accuracy of PSNR in predicting video quality for different video scenes and frame rates. Telecommun. Syst..

[13] Wang Z., Bovik A.C., Sheikh H.R., Simoncelli E.P. (2004). Image quality assessment: From error visibility to structural similarity. IEEE Trans. Image Process..

[14] He K., Sun J., Tang X. (2012). Guided image filtering. IEEE Trans. Pattern Anal. Mach. Intell..

[15] Brownrigg D.R. (1984). The weighted median filter. Commun. ACM.

[16] Ma Z., He K., Wei Y., Sun J., Wu E. Constant time weighted median filtering for stereo matching and beyond. Proceedings of the IEEE International Conference on Computer Vision (ICCV).

[17] Hosni A., Bleyer M., Rhemann C., Gelautz M., Rother C. Real-time local stereo matching using guided image filtering. Proceedings of the IEEE International Conference on Multimedia and Expo (ICME).

[18] Chen R., Han S., Xu J., Su H. Point-based multi-view stereo network. Proceedings of the IEEE International Conference on Computer Vision (ICCV).

[19] Manuel M.G.V., Manuel M.M.J., Edith M.M.N., Ivone R.A.P., Ramirez S.R.E. Disparity map estimation with deep learning in stereo vision. Proceedings of the Regional Consortium for Foundations, Research and Spread of Emerging Technologies in Computing Sciences (RCCS+SPIDTEC2).

[20] Su H., Maji S., Kalogerakis E., Learned-Miller E. Multi-view convolutional neural networks for 3D shape recognition. Proceedings of the IEEE International Conference on Computer Vision (ICCV).

[21] Kuhn A., Sormann C., Rossi M., Erdler O., Fraundorfer F. DeepC-MVS: Deep confidence prediction for Multi-view stereo reconstruction. Proceedings of the International Conference on 3D Vision (3DV).

[22] Kuhn A., Lin S., Erdler O. Plane completion and filtering for multi-view stereo reconstruction. Proceedings of the German Conference on Pattern Recognition (GCPR).

[23] Wang K., Shen S. Mvdepthnet: Real-time multiview depth estimation neural network. Proceedings of the International Conference on 3D Vision (3DV).

[24] Huang P.H., Matzen K., Kopf J., Ahuja N., Huang J.B. Deepmvs: Learning multi-view stereopsis. Proceedings of the IEEE Conference on Computer Vision and Pattern Recognition (CVPR).

[25] Im S., Jeon H.G., Lin S., Kweon I.S. Dpsnet: End-to-end deep plane sweep stereo. Proceedings of the International Conference on Learning Representations (ICLR).

[26] Wu G., Li Y., Huang Y., Liu Y. (2019). Joint view synthesis and disparity refinement for stereo matching. Front. Comput. Sci..

[27] Zhou T., Tucker R., Flynn J., Fyffe G., Snavely N. (2018). Stereo magnification: Learning view synthesis using multiplane images. ACM Trans. Graph..

[28] Flynn J., Broxton M., Debevec P., DuVall M., Fyffe G., Overbeck R., Snavely N., Tucker R. Deepview: View synthesis with learned gradient descent. Proceedings of the IEEE/CVF Conference on Computer Vision and Pattern Recognition (CVPR).

[29] Srinivasan P.P., Tucker R., Barron J.T., Ramamoorthi R., Ng R., Snavely N. Pushing the boundaries of view extrapolation with multiplane images. Proceedings of the IEEE Computer Society Conference on Computer Vision and Pattern Recognition (CVPR).

[30] Kanade T., Okutomi M. (1994). A stereo matching algorithm with an adaptive window: Theory and experiment. IEEE Trans. Pattern Anal. Mach. Intell..

[31] Zabih R., Woodfill J. Non-parametric local transforms for computing visual correspondence. Proceedings of the European conference on computer vision (ECCV).

[32] Schönberger J.L., Zheng E., Frahm J.M., Pollefeys M. Pixelwise view selection for unstructured multi-view stereo. Proceedings of the European Conference on Computer Vision (ECCV).

[33] Middlebury Stereo Evaluation-Version 2. https://vision.middlebury.edu/stereo/eval/.

[34] Huang X., Yuan C., Zhang J. A systematic stereo matching framework based on adaptive color transformation and patch-match forest. J. Vis. Commun. Image Represent.

[35] Li H., Gao Y., Huang Z., Zhang Y. (2019). Stereo matching based on multi-scale fusion and multi-type support regions. J. Opt. Soc. Amer. A. JOSAA.

[36] Wu W., Zhu H., Yu S., Shi J. (2019). Stereo matching with fusing adaptive support weights. IEEE Access.

[37] Besse F., Rother C., Fitzgibbon A., Kautz J. (2013). PMBP: PatchMatch belief propagation for correspondence field estimation. Int. J. Comput. Vis..

[38] Li Y., Min D., Brown M.S., Do M.N., Lu J. SPM-BP: Sped-up PatchMatch belief propagation for continuous MRFs. Proceedings of the IEEE International Conference on Computer Vision (ICCV).

[39] Taniai T., Matsushita Y., Naemura T. Graph cut based continuous stereo matching using locally shared labels. Proceedings of the IEEE/CVF Conference on Computer Vision and Pattern Recognition (CVPR).

[40] Mei X., Sun X., Dong W., Wang H., Zhang X. Segment-tree based cost aggregation for stereo matching. Proceedings of the IEEE Conference on Computer Vision and Pattern Recognition (CVPR).

[41] Taniai T., Matsushita Y., Sato Y., Naemura T. (2017). Continuous 3D label stereo matching using local expansion moves. IEEE Trans. Pattern Anal. Mach. Intell..

[42] Bleyer M., Rhemann C., Rother C. PatchMatch stereo–stereo matching with slanted support windows. http://www.bmva.org/bmvc/2011/proceedings/paper14/paper14.pdf.

[43] Li L., Zhang S., Yu X., Zhang L. (2016). PMSC: Patchmatch-based superpixel cut for accurate stereo matching. IEEE Trans. Circuits Syst. Video Technol..

[44] Yang Q., Ji P., Li D., Yao S., Zhang M. (2014). Fast stereo matching using adaptive guided filtering. Image Vis. Comput..

[45] Yang Q. (2015). Stereo matching using tree filtering. IEEE Trans. Pattern Anal. Mach. Intell..

[46] Zhang K., Fang Y., Min D., Sun L., Yang S., Yan S. (2017). Cross-scale cost aggregation for stereo matching. IEEE Trans. Circuits Syst. Video Technol..

[47] Hamzah R.A., Kadmin A.F., Hamid M.S., Ghani S.F.A., Ibrahim H. (2018). Improvement of stereo matching algorithm for 3D surface reconstruction. Signal Process. Image Commun..

[48] Luo G., Zhu Y. (2016). Foreground removal approach for hole filling in 3D video and view synthesis. IEEE Trans. Circuits Syst. Video Technol..

[49] Oliveira A.Q., Walter M., Jung C.R. (2018). An artifact-type aware dibr method for view synthesis. IEEE Signal Process. Lett..

[50] Jain A.K., Tran L.C., Khoshabeh R., Nguyen T.Q. Efficient stereo-to-multiview synthesis. Proceedings of the IEEE International Conference on Acoustics, Speech and Signal Processing (ICASSP).

[51] Tran L.C., Bal C., Pal C.J., Nguyen T.Q. (2012). On consistent inter-view synthesis for autostereoscopic displays. 3D Res..

[52] Ramachandran G., Rupp M. Multiview synthesis from stereo views. Proceedings of the International Conference on Systems, Signals and Image Processing (IWSSIP).

[53] Zheng E., Dunn E., Jojic V., Frahm J.M. Patchmatch based joint view selection and depthmap estimation. Proceedings of the IEEE Conference on Computer Vision and Pattern Recognition (CVPR).

[54] Hu X., Mordohai P. Least commitment, viewpoint-based, multi-view stereo. Proceedings of the International Conference on 3D Imaging, Modeling, Processing, Visualization and Transmission (3DIMPVT).

[55] Furukawa Y., Ponce J. (2009). Accurate, dense, and robust multiview stereopsis. IEEE Trans. Pattern Anal. Mach. Intell..

[56] Zaharescu A., Boyer E., Horaud R. (2010). Topology-adaptive mesh deformation for surface evolution, morphing, and multiview reconstruction. IEEE Trans. Pattern Anal. Mach. Intell..

[57] Tylecek R., Šára R. (2010). Refinement of surface mesh for accurate multi-view reconstruction. Int. J. Virtual Real..

[58] Jancosek M., Pajdla T. Multi-view reconstruction preserving weakly-supported surfaces. Proceedings of the IEEE Conference on Computer Vision and Pattern Recognition (CVPR).

[59] Galliani S., Lasinger K., Schindler K. Massively parallel multiview stereopsis by surface normal diffusion. Proceedings of the IEEE International Conference on Computer Vision (ICCV).

[60] ETH3D Low-Resolution Many-View Benchmark. https://www.eth3d.net/low_res_many_view.

[61] Xue T., Owens A., Scharstein D., Geosele M., Szeliski R. (2019). Multi-frame stereo matching with edges, planes, and superpixels. Image Vis. Comput..

[62] Chang J.R., Chen Y.S. Pyramid stereo matching network. Proceedings of the IEEE Conference on Computer Vision and Pattern Recognition (CVPR).

[63] Park I.K. (2018). Deep self-guided cost aggregation for stereo matching. Pattern Recognit. Lett..

[64] Ma N., Men Y., Men C., Li X. (2016). Accurate dense stereo matching based on image segmentation using an adaptive multi-cost approach. Symmetry.

[65] Yin J., Zhu H., Yuan D., Xue T. (2017). Sparse representation over discriminative dictionary for stereo matching. Pattern Recognit..

[66] Hamzah R.A., Ibrahim H., Hassan A.H.A. (2017). Stereo matching algorithm based on per pixel difference adjustment, iterative guided filter and graph segmentation. J. Vis. Commun. Image Represent..

[67] Zhang K., Li J., Li Y., Hu W., Sun L., Yang S. Binary stereo matching. Proceedings of the International Conference on Pattern Recognition (ICPR).

[68] Bricola J.C., Bilodeau M., Beucher S. Morphological Processing of Stereoscopic Image Superimpositions for Disparity Map Estimation. https://hal.archives-ouvertes.fr/hal-01330139/.

[69] Kitagawa M., Shimizu I., Sara R. High accuracy local stereo matching using DoG scale map. Proceedings of the Fifteenth IAPR International Conference on Machine Vision Applications (MVA).

[70] Mao W., Gong M. Disparity filtering with 3D convolutional neural networks. Proceedings of the 15th Conference on Computer and Robot Vision (CRV).

[71] Hirschmuller H. (2008). Stereo processing by semiglobal matching and mutual information. IEEE Trans. Pattern Anal. Mach. Intel. TPAMI.

[72] Zbontar J., LeCun Y. (2016). Stereo matching by training a convolutional neural network to compare image patches. J. Mach. Learn. Res. JMLR.

